# Effect of Dexmedetomidine-Assisted Intravenous Inhalation Combined Anesthesia on Cerebral Oxygen Metabolism and Serum Th1/Th2 Level in Elderly Colorectal Cancer Patients

**DOI:** 10.3389/fsurg.2021.832646

**Published:** 2022-01-25

**Authors:** Yixun Tang, Jitong Liu, Xiaoling Huang, Huijuan Ding, Suhong Tan, Yimin Zhu

**Affiliations:** ^1^Department of Anesthesiology, Hunan Provincial People's Hospital, The First Affiliated Hospital of Hunan Normal University, Changsha, China; ^2^School of Life Sciences, Hunan Normal University, Changsha, China; ^3^Clinical Research Center for Anesthesiology of ERAS in Hunan Province, Changsha, China; ^4^Hunan Provincial Key Laboratory of Emergency and Critical Care Metabonomics, Institute of Emergency Medicine, Hunan Provincial People's Hospital, The First Affiliated Hospital of Hunan Normal University, Changsha, China

**Keywords:** elderly patients with colorectal cancer, dexmedetomidine, cerebral oxygen metabolism, cognitive impairment, immune function

## Abstract

**Objective:**

To observe the effect of dexmedetomidine-assisted intravenous inhalation combined anesthesia on cerebral oxygen metabolism and serum Th1/Th2 levels in elderly patients with colorectal cancer.

**Method:**

From April 2018 to May 2020,100 elderly patients undergoing elective laparoscopic radical resection of colorectal cancer were prospectively selected and randomly divided into observation group and control group. Before induction of anesthesia, the loading dose of dexmedetomidine was given at 0.5 μg/kg, and the infusion time was 15 min. After tracheal intubation, 0.4 μg/kg/h dexmedetomidine was continuously pumped, and the infusion was stopped 40 min before the end of the operation. In the control group, the same amount of 0.9% sodium chloride was injected intravenously in the same way. 30 min before induction of anesthesia (T_0_), immediately before induction of anesthesia (T_1_), immediately after tracheal intubation (T_2_), 40 min before operation (T_3_), and immediately after operation (T_4_), record the blood oxygen content of the artery and internal jugular vein Difference (D(a-jv)O_2_), brain oxygen uptake rate (COER%), brain oxygen saturation (rSO_2_) mean. VAS scale, Ramsay scale, MoCA scale were taken at 6, 12, 24, and 48 h postoperatively to evaluate analgesia, sedation, and cognitive function. And monitor the levels of interferon-γ (IFN-γ), interleukin-4 (IL-4), myelin basic protein (MBP), neuron-specific enolase (NSE) and S100β. The occurrence of restlessness and adverse reactions during the recovery period of the two groups were compared.

**Result:**

The levels of D(a-jv)O_2_, COER%, and rSO_2_ in the control group and observation group were higher than the preoperative basic values at T2, T3, and T4 (*P* < 0.05); The levels of D(a-jv)O_2_, COER%, and rSO_2_ in the observation group were lower than those in the control group at T_2_, T_3_, and T_4_ (*P* < 0.05). The VAS score and Ramsay score of the observation group were lower than those of the control group at 6, 12, 24, and 48 h after surgery, while the MoCA score was higher than that of the control group (*P* < 0.05). In addition, the serum IFN-γ, MBP, NSE and S100β levels of the observation group were lower than those of the control group (*P* < 0.05), and the ratio of IFN-γ/IL-4 was higher than that of the control group (*P* < 0.05). The overall incidence of adverse reactions in the observation group was lower than that in the control group [32.0% (16/50) vs. 12.0% (6/50), *P* < 0.05].

**Conclusion:**

Dexmedetomidine-assisted combined intravenous and inhalation anesthesia is beneficial to reduce perioperative cerebral oxygen metabolism and improve postoperative immunosuppression in elderly patients with colorectal cancer. It has a certain protective effect on nerve injury after operation, thus improving the cognitive function of patients and reducing the occurrence of adverse reactions.

## Introduction

Colorectal cancer is one of the high-risk gastrointestinal malignant tumors, and more than 50% of patients die, ranking the third in malignant tumor deaths. Laparoscopic radical surgery is the main treatment of colorectal cancer, which greatly reduces the surgical trauma and ensures the smooth progress of postoperative rehabilitation. However, laparoscopic surgery establishes artificial pneumoperitoneum, and a large amount of CO_2_ enters the peripheral blood circulation through the peritoneum, which affects brain metabolism and leads to nerve damage. Elderly patients have decreased body function and organ reserve function, and they are more likely to suffer from acute lung injury and postoperative cognitive dysfunction when receiving surgical treatment. Elderly patients are prone to cognitive dysfunction or delirium after surgery, and the selection of anesthetic methods and drugs is an important factors affecting the injury of brain neurons (1). Dexmedetomidine is an α2 receptor high-affinity agonist, especially for the α2A receptor located in the locus coeruleus nucleus in the brain, which has high selectivity. By inhibiting the release of sympathetic nerve excitatory transmitters and the upward transmission of peripheral pain signals, Produce sedation and analgesia (2). In addition, dexmedetomidine has a certain affinity for imidazoline I1 receptors located in brain stem and hippocampus, which inhibits the release of catecholamine and excitotoxic amino acids, reduces the sensitivity of neurons to glutamic acid, and thus plays a role in brain protection (3). At present, dexmedetomidine has been used in neurosurgery, thoracic surgery, cardiology surgery, pediatric surgery and other operations, but there are still few studies in the operation of abdominal tumor patients. Malignant tumor patients generally have disorders of immune system. Surgery and anesthesia may further inhibit the cellular immune function of the body, thus affecting the prognosis of patients (4). Dexmedetomidine is a commonly used auxiliary anesthetic in surgery, which has the effects of central nervous protection and cardiopulmonary function protection. This study was designed to observe the effects of dexmedetomidine combined with intravenous inhalation anesthesia on brain oxygen metabolism and serum Th1/Th2 levels in elderly patients with colorectal cancer, aiming to provide certain evidence-based evidence and reference for the clinical application of dexmedetomidine.

## Clinical Data and Methods

### The Clinical Data Selected

From April 2018 to May 2020,100 elderly patients who underwent elective laparoscopic radical resection of colorectal cancer in the First Affiliated Hospital of Hunan Normal University were taken as the overall research object. Combined with clinical manifestations, related imaging manifestations and pathological first-stage biopsy, rectal cancer was diagnosed. Inclusion criteria: age 60–85 years old; Indications for laparoscopic radical resection of rectal cancer; American society of anesthesiologists (ASA) (5) grade I-II; There is no obvious contraindication to anesthesia. Exclusion criteria: those who have a history of neurological or mental illness; People with a history of drug allergy or long-term alcoholism; Those who took anticoagulants, sedatives, antidepressants, non-steroidal anti-inflammatory drugs or hormone drugs 3 months before operation; Eliminate those whose blood loss during the operation is > 20% of the basal blood volume; Those with intractable hypotension or anaphylactic shock. With the approval of the ethics committee of this hospital and the informed consent of the patients, 100 patients were divided into control group and observation group according to equal-length random sampling method with the approval of the ethics committee of the hospital and the informed consent of the patients. Comparing the age, gender composition, weight, body mass index (BMI), ASA classification, and cancer type of the two groups of patients, the difference was not statistically significant (*P* > 0.05) and was comparable. As shown in Table 1.

**Table 1:** Comparison of the general conditions of the two groups of patients ($\bar{x}$ ± s).

**Group**	***n***	**Gender**		**Age (year)**	**BMI (kg/m^**2**^)**	**ASA**		**Type**		**TNM stage**		
		**Male**	**Female**			**I**	**II**	**Colon cancer**	**Rectal cancer**	**I**	**II**	**III**
Control group	50	38	12	70.68 ± 6.48	23.25 ± 1.38	24	26	34	16	19	26	5
Observation group	50	30	20	69.70 ± 6.58	22.75 ± 1.49	31	19	28	22	14	27	9
*t* value	-	2.941		0.750	1.741	1.980		1.528		1.919		
*P* value	-	0.086		0.455	0.085	0.159		0.216		0.383		

### Methods of Anesthesia

Routine fasting and drinking before operation lasted 6–8 h. After entering the room, open the venous channel to monitor the electrocardiogram, mean artery pressure (MAP), heart rate (HR), pulse oxygen saturation (SpO_2_), and bispectral index (BIS). Patients in the observation group were given dexmedetomidine (Jiangsu Hengrui Pharmaceutical Co., Ltd., specification: 2 mL: 200 μg, batch number 190423BP) loading dose of 0.5 μg/kg before induction of anesthesia, and the infusion time was 20 min. After tracheal intubation, dexmedetomidine was continuously pumped at 0.4 μg/kg/h (prepared with normal saline to 4 μg/mL) until 40 min before the end of the operation. Patients in the control group were given an equal volume of 0.9% sodium chloride injection intravenously in the same way General anesthesia induction: intravenous infusion of propofol (1.5–2 mg/kg), after the patient's consciousness disappears, slow intravenous bolus of midazolam (0.02 mg/kg) and sufentanil citrate (2.0–3.5 μg/kg), etomidate (0.2 mg/kg) and cis-atracurium benzenesulfonate (0.1–0.2 mg/kg), the patients were subjected to sequential induction of general anesthesia; After the muscle relaxation takes effect, perform tracheal intubation, connect to anesthesia machine for mechanical ventilation, tidal volume 6–8 mL/kg, frequency 12–15 times/min, continuous inhalation of sevoflurane 1%-2%, target-controlled infusion C Poofol (4–10 mg/kg/h) was used for anesthesia maintenance, intermittent intravenous injection of cis-atracurium to relax the muscles. BIS is maintained between 40 and 60. Connect the intravenous analgesic pump after the operation. Patient controlled intravenous analgesia (PCIA) was used, dexmedetomidine (0.5 g/kg) + sufentanil (0.8 g/kg) was used, and normal saline was diluted into 100 ml solution. The dose is 1 mL, and the lock time is 15 min.

### Observation Indicators

#### Cerebral Oxygen Metabolism

30 Minutes Before Induction of Anesthesia (T_0_), Immediately Before Induction of Anesthesia (T_1_), Immediately After Tracheal Intubation (T_2_), 40 Min Before the Operation (T_3_), and Immediately After the Operation (T_4_), Collect Blood Samples From the Radial Artery and Internal Jugular Venous Bulb, and Calculate the Arterial-Internal Jugular Venous Blood Oxygen Difference (D_(a−Jv)_O_2_) and Cerebral Oxygen Uptake Rate (COER%) According to the Fick Formula (6). COER% = [(CaO_2_-CvO_2_)/CaO_2_] × 100%, CaO_2_ Is Arterial Blood Oxygen Content, CvO_2_ Is Arterial Oxygen Consumption. At the Same Time, a Near-Infrared Photometer Was Used to Monitor the Mean Value of Cerebral Oxygen Saturation (RSO2) on Both Sides of the Frontal Line.

#### Postoperative Analgesia, Sedation Effect and Early Postoperative Cognitive Function

At 6, 12, 24, and 48 h after surgery, the visual analog scale (VAS) method was used to evaluate the analgesic effect. VAS scoring criteria: 0 point means no pain, 10 points means the most pain, < 3 points means good analgesia, and ≥5 points means poor analgesic effect. Ramsay score was used to evaluate the sedative effect. The total Ramsay score was 6 points. The higher the score was, the higher the degree of sedation would be. Montreal Cognitive Assessment (MoCA) score was also recorded. The MoCA scale included visual space and executive function, naming, memory, attention, language, abstraction, delayed memory and orientation, with a full score of 30. A lower score indicated lower cognitive function, < 26 indicated abnormal.

#### Postoperative Laboratory Index Monitoring

Serum samples were collected at the preoperative basic value and immediately after the operation, 6, 12, 24, and 48 h after the operation, and the ELISA kit was used to monitor interferon-γ (IFN-γ) and interleukin-4 (interleukin-4, IL-4), myelin basic protein (MBP), neuron-specific enolase (NSE), S100β content. The kit used was purchased from Shanghai Kanglang Biotechnology Co., Ltd.

#### Adverse Reactions

The incidence of restlessness, dizziness, nausea and vomiting, and respiratory depression in the two groups of patients during the recovery period was counted within 48 h after the operation.

### Statistical Processing

Using SPSS19.0 statistical software, measurement data are expressed as mean ± standard deviation ($\bar{x}$ ±s), using repeated measures analysis of variance for intra-group comparison, and comparison between groups using LSD-*t* test; The count data is expressed as a percentage or rate (%), and the χ^2^ test is performed. *P* < 0.05 indicates that the difference is statistically significant.

## Results

### Comparison of Surgical Conditions Between the Two Groups

There was no significant difference in anesthesia induction time, operation time, anesthesia maintenance time, fluid replacement, blood loss during operation, directional recovery time, extubation time and observation time in PACU after anesthesia (*P* > 0.05). As shown in Table 2.

**Table 2:** Comparison of surgical conditions between the two groups ($\bar{x}$ ± s).

**Group**	**n**	**Anesthesia induction time (min)**	**Operation time (min)**	**Anesthesia maintenance time (min)**	**Rehydration volume (L)**	**Volume of blood loss (mL)**	**Orientation recovery time (min)**	**Extubation time (min)**
Control group	50	10.94 ± 2.32	145.78 ± 43.27	163.84 ± 30.59	1.92 ± 0.73	389.76 ± 54.63	16.26 ± 4.58	20.48 ± 5.67
Observation group	50	12.06 ± 3.75	161.52 ± 48.39	172.63 ± 28.95	2.07 ± 0.78	402.14 ± 64.85	15.44 ± 5.37	19.16 ± 5.52
*t-*value	-	1.796	1.715	1.476	0.993	1.032	0.822	1.180
*P-*value	-	0.076	0.090	0.143	0.323	0.304	0.413	0.241

### Changes in Cerebral Oxygen Metabolism Indexes of the Two Groups

**Figure 1:**
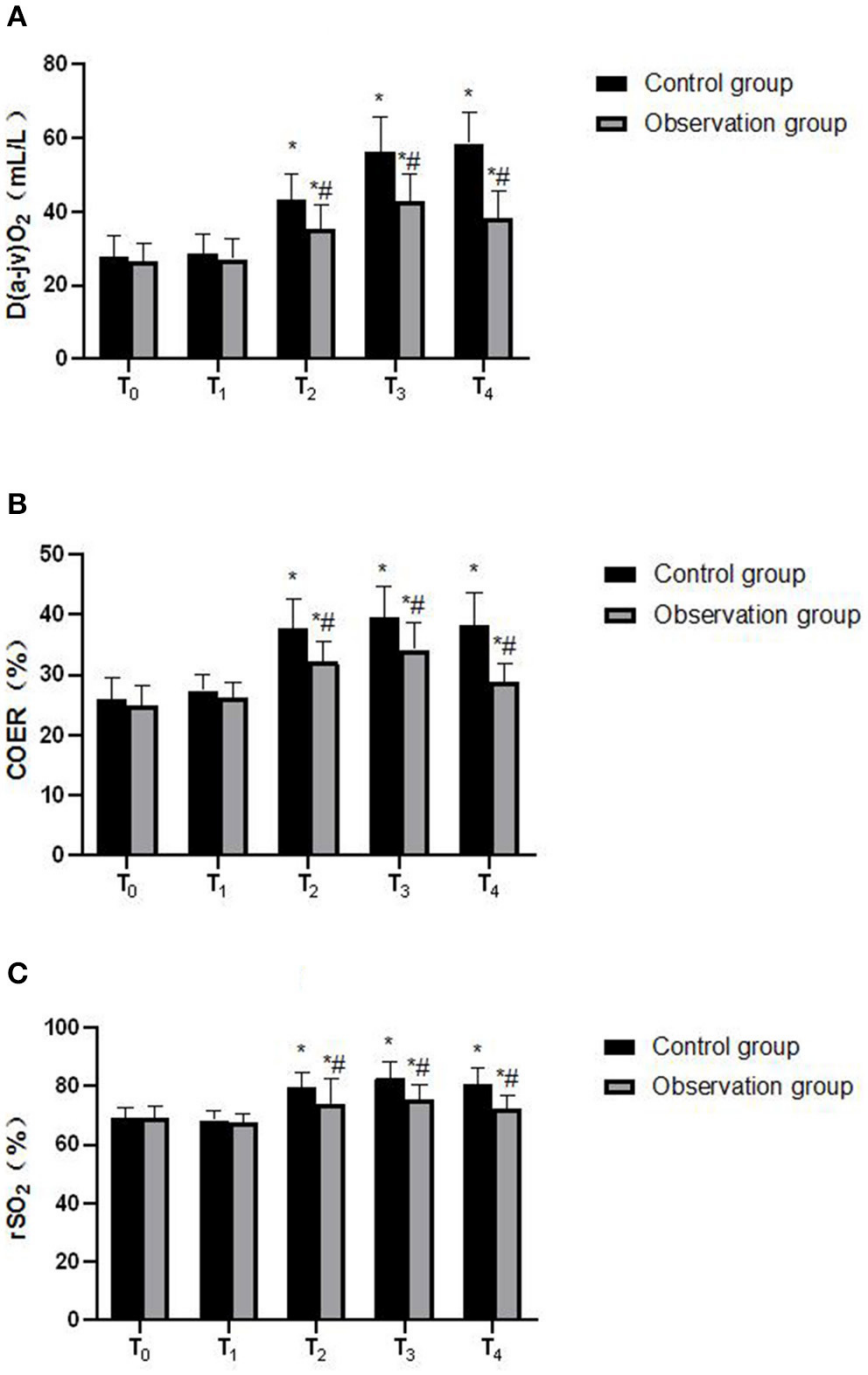
Comparison of D(a-jv)O2, COER%, rSO2 at different time points between the two groups. **(A)** D(a-jv)O2; **(B)** COER% and **(C)** rSO2. Compared with T_0_ in the samegroup, *P < 0.05; compared with control group, ^#^*P* < 0.05.

The analysis of variance with repeated measures design was used to compare the levels of D_(a−jv)_O_2_, COER%, and rSO_2_ at each time point of T_0_-T_1_ between the two groups. ①D_(a−jv)_O_2_, COER%, rSO_2_ levels were statistically different at different time points (*F* = 17.241, 8.260, 4.385, *P* < 0.05), the control group and observation group patients D_(a−jv)_O_2_ COER% and rSO_2_ levels at T_2_, T_3_, and T_4_ were higher than the preoperative basic value (*P* < 0.05). ②The observation group and the control group had statistical differences in D_(a−jv)_O_2_, COER%, rSO_2_ levels (*F* = 16.451, 5.638, 8.799, *P* < 0.05). The levels of D_(a−jv)_O_2_, COER% and rSO_2_ of the observation group were lower than those of the control group at T_2_, T_3_, and T_4_ (*P* < 0.05). ③There was a statistical difference in the trend of changes in the levels of D_(a−jv)_O_2_, COER%, and rSO_2_ between the two groups of patients (*F* = 38.757, 12.605, 9.374, *P* < 0.05). As shown in Figure 1.

### Comparison of Postoperative Analgesia, Sedation Effects and Cognitive Function Between the Two Groups

**Figure 2:**
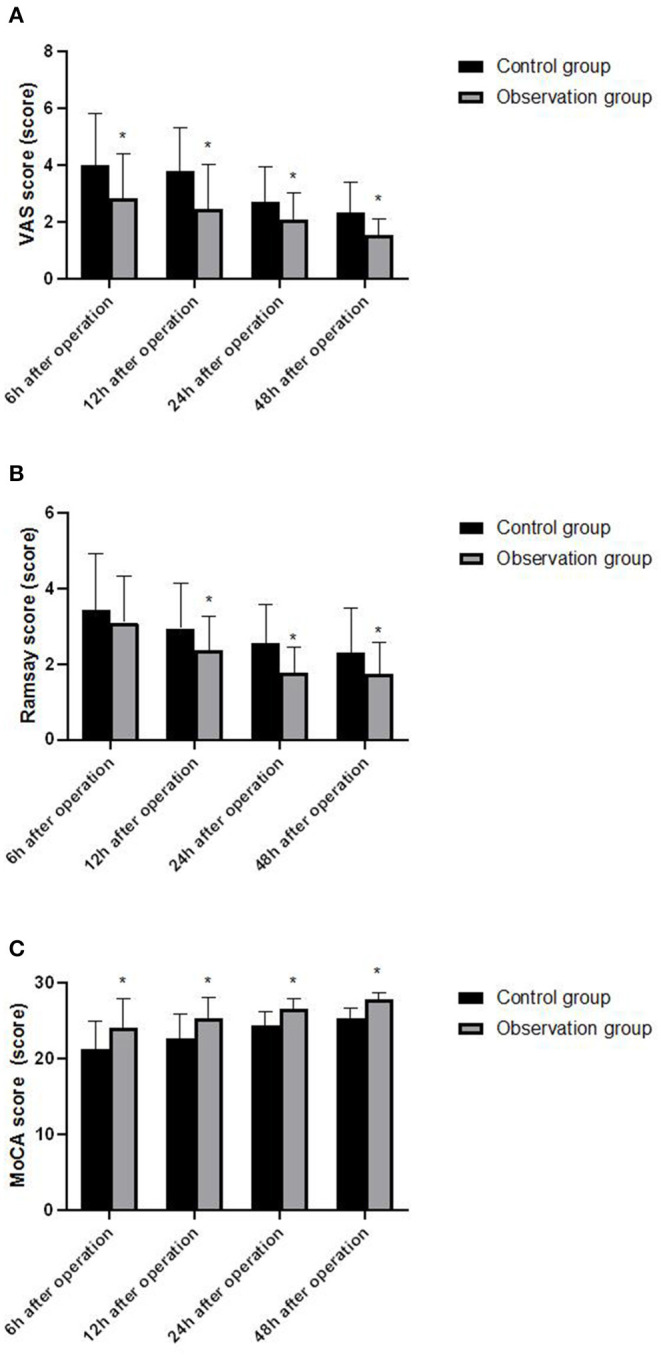
Comparison of VAS score, Ramsay score and MoCA score between the two groups of patients at different time points. **(A)** VAS score; **(B)** Ramsay score and **(C)** MoCA score. Compared with the control group, *P < 0.05.

The analysis of variance with repeated measures design was used to compare the VAS score, Ramsay score, and MoCA score at each time point of 6, 12, 24, and 48 h after the operation of the two groups. 1. VAS score, Ramsay score, and MoCA score were statistically different at different time points (*F* = 12.351, 5.462, 8.291, *P* < 0.05). 2. There was a statistical difference between the observation group and the control group in VAS score, Ramsay score, and MoCA score (*F* = 21.358, 27.642, 13.058, *P* < 0.05). The observation group had VAS score and Ramsay score at 6, 12, 24, 48 h were lower than the control group, while the MoCA score was higher than the control group (*P* < 0.05). 3. There was a statistical difference in the trend of VAS score, Ramsay score, and MoCA score between the two groups (*F* = 42.637, 27.814, 29.854, *P* < 0.05). As shown in Figure 2.

### Comparison of Laboratory Indicators Between the Two Groups

**Figure 3:**
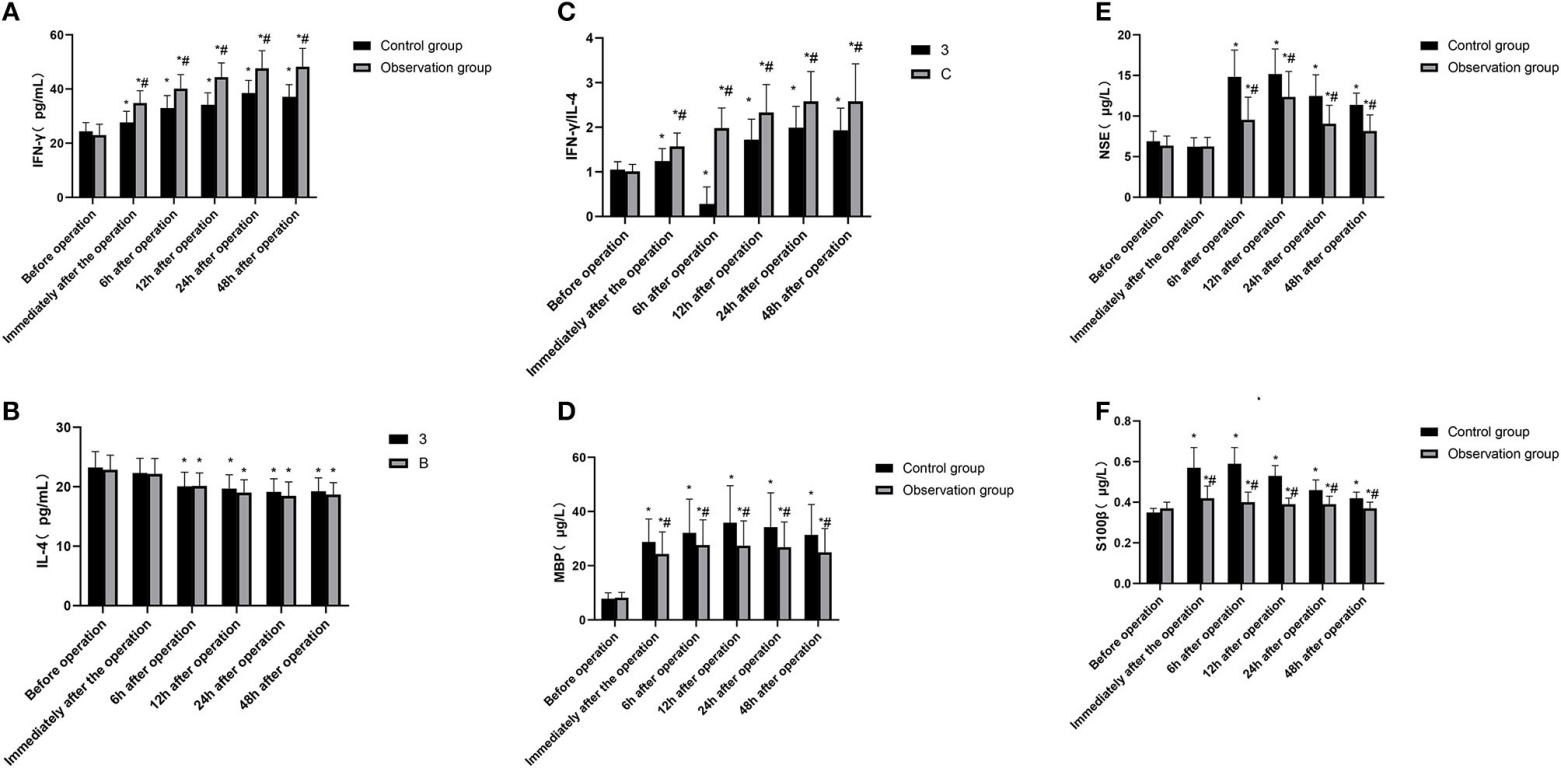
Comparison of IFN-γ, IL-4, IFN-γ/IL-4, neurological impairment related indexes between the two groups of patients at different time points. **(A)** IFN-γ; **(B)** IL-4; **(C)** IFN-γ/IL-4; **(D)** MBP; **(E)** NSE and **(F)** S100β. Compared with the preoperative basic value of the same group, *P < 0.05; compared with the control group, ^#^*P* < 0.05.

The analysis of variance with repeated measures design was used to compare the serum IFN-and ILIL-4els and IFN - γ/IL - 4 ratios of the two groups of patients before the operation, immediately after the operation, and at each time point of 6, 12, 24, and 48 h after the operation. 1. There were statistical differences in serum IFN-γ levels, IFN-γ/IL-4 ratio, serum MBP, NSE and S100β levels at different time points (*F* = 8.410, 28.573, 37.258, 19.267, 4.851, *P* < 0.05). There was no statistical difference in serum IL-4 levels at time points (*F* = 2.059, *P* = 0.148). 2. There were statistical differences in serum IFN-γ levels, IFN-γ/IL-4 ratio, serum MBP, NSE and S100β levels between the observation group and the control group (*F* = 13.936, 37.815, 7.824, 9.673, 5.897, *P* < 0.05). The serum IFN-γ levels, MBP and S100β levels of the observation group were lower than those of the control group immediately after the operation and at 6, 12, 24, and 48 h after the operation (*P* < 0.05). At the same time, IFN-γ The ratio of /IL-4 was higher than that of the control group (*P* < 0.05). Serum NSE levels in the observation group were lower than those in the control group at 6, 12, 24, and 48 h after surgery (*P* < 0.05). In addition, there was no significant difference in serum IL-4 levels between the two groups of patients (*F* = 1.734, *P* = 0.325). 3. There were statistical differences in the changes of serum IFN-γ levels, IFN-γ/IL-4 ratio, serum MBP, NSE and S100β levels between the two groups (*F* = 43.218, 786.454, 118.379, 72.361, 28.504, *P* < 0.05), while the comparison of the trend of changes in serum IL-4 levels between the two groups of patients, the difference was not statistically significant (*F* = 0.927, *P* = 0.564). As shown in Figure 3.

### Comparison of Postoperative Adverse Reactions Between the Two Groups

**Figure 4:**
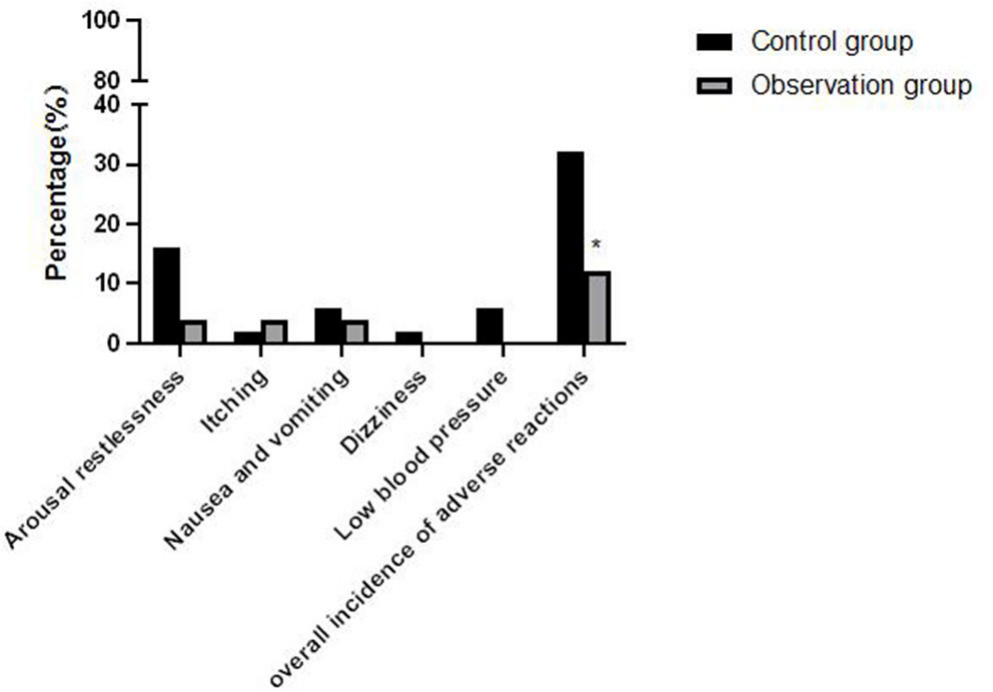
Comparison of the incidence of adverse reactions between the two groups of patients. Compared with control group, *P < 0.05.

All patients successfully completed the tracheal intubation, and no coughing or body odor movement occurred. The total incidence of adverse reactions (including restlessness in convalescence) in the control group and observation group was 32.0% (16/50) and 12.0% (6/50), respectively. The difference was statistically significant after the χ^2^ test (χ^2^ = 5.828, *P* = 0.016), the overall incidence of adverse reactions in the observation group was slightly lower than that in the control group. As shown in Figure 4.

## Discussion

Postoperative cognitive dysfunction (POCD) refers to symptoms such as mental confusion, degeneration of mental function, and decreased social ability within a few days or months after surgery (7), It is more common in patients undergoing major surgery such as cardiology, gastrointestinal surgery, trauma surgery, and tumor surgery. A small number of patients can even persist for a long time, It greatly increases the risk of postoperative complications and prolongs the hospital stay, which in turn affects the patient's prognosis and postoperative quality of life.

Old age is the risk factor that is closely related to POCD disease Scholars such as Czyz-Szypenbejl (8) found that the incidence of POCD in elderly patients over 60 years old within 7 days was about 17 ~ 56%, and with the increase of age, the incidence of POCD increased. The incidence of POCD in elderly patients is higher than that of patients aged 60 to 69, and the proportion of patients with long-term persistent POCD has also increased greatly, and some patients may even progress to Alzheimer's disease. It is speculated that the principle lies in the degenerative changes in the brain structure and function of the elderly, the aging of neuronal nuclei, the increase in apoptosis rate, the decrease in neurotransmitter secretion, the deterioration of brain glucose metabolism and neural signal transmission functions, which makes the elderly population The brain is more susceptible to damage from anesthetics (9).

Besides age, anesthesia methods and narcotic drugs are also important factors that affect postoperative cognitive function. According to a multi-center study conducted by Wang Dongting and other scholars (10), 400 elderly patients who were to undergo radical resection of colorectal cancer were followed up and observed. MoCA score is a common cognitive function screening tool, which is used as an auxiliary diagnostic method for POCD disease. The results showed that the incidence of POCD was about 15.6 %, and the duration of anesthesia and the proportion of general anesthesia are independent risk factors for the occurrence of POCD. In this study, the enrolled patients with colorectal cancer all used propofol-sevoflurane intravenous inhalation combined with general anesthesia. Propofol is a highly fat-soluble intravenous anesthetic. Its sedative mechanism and enhancement of γ-amino Butyrate A receptor activity, which in turn enhances the inhibitory postsynaptic current, is related (11). Gamma-aminobutyric acid receptor activity can lead to long-term potentiation (LTP) that inhibits the excitatory transmission of pyramidal neurons in the hippocampal CA 1 region, and then affect the learning and cognitive functions of patients (12). In addition, scholars such as Alkire MT (13) confirmed that inhalational anesthetics, such as sevoflurane and sevoflurane at the relative minimum alveolar concentration, within 24 h, the memory loss of nitrous oxide is more serious, but it is compared with other inhaled anesthetics. Sexual anesthetics, sevoflurane is metabolized quickly and has little effect on the nervous system, but it still cannot avoid the damage to the cognitive function of elderly patients after surgery.

Dexmedetomidine is an α 2 adrenergic receptor agonist with high selectivity and specificity. By inhibiting the activity of sympathetic nerves and the secretion of stress hormones such as cortisol and catecholamine, it can reduce the damage to hippocampus, thus reducing the influence of anesthetics on the early postoperative cognitive function of elderly patients (14). In this study, patients in the observation group were given dexmedetomidine-assisted intravenous inhalation combined anesthesia, and the postoperative VAS score and Ramsay score were significantly lower than those of the control group, indicating that dexmedetomidine-assisted combined intravenous inhalation anesthesia has good analgesia and sedation. In addition, the postoperative MoCA score of the observation group was higher than that of the control group, and the levels of D_(a−jv)_O_2_, COER%, and rSO_2_ were lower than those of the control group. D_(a−jv)_O_2_, COER%, and rSO_2_ reflect brain oxygen An important indicator of metabolism and blood supply of brain tissue. If the oxygen uptake rate of brain decreases, the oxygen supply is greater than the oxygen consumption, indicating that the brain tissue has a normal aerobic metabolism. Therefore, the above results indicate that dexmedetomidine can effectively maintain the balance of cerebral oxygen supply and demand in elderly colorectal cancer surgery. In addition, MBP, NSE, S100β and other indicators are important marker molecules that reflect the damage of nerve cells, and have a certain effect on maintaining the proliferation and differentiation of neurons or glial cells (15). In this study, the levels of MBP, NSE, and S100β in the observation group were significantly lower than those in the control group, indicating that dexmedetomidine has a certain protective effect on nerve function damage caused by intravenous inhalation and combined anesthesia in elderly patients with colorectal cancer. Elderly patients with colorectal cancer often have autoimmune disorders, as well as surgical trauma, anesthesia stimulation, and postoperative stress, which further inhibit the body's humoral immune function (1, 16). Immune dysfunction is also one of the important reasons leading to cognitive dysfunction in patients (17). In this study, the postoperative IFN-γ/IL-4 ratio of the observation group was higher than that of the control group. IFN-γ and IL-4 were the most typical cytokines of Th1 and Th2 cells, and the ratio of IFN-γ/IL-4 increased Gao shows that under the action of dexmedetomidine, the Th1/Th2 response pattern gradually drifts to Th1 cells, which helps the recovery of the patient's immune function after surgery.

n summary, dexmedetomidine-assisted intravenous inhalation combined anesthesia is beneficial to reduce perioperative cerebral oxygen metabolism in elderly patients with colorectal cancer, improve postoperative immunosuppression status, and has a certain protective effect on postoperative neurological damage. In order to improve patients' cognitive function and reduce the occurrence of adverse reactions, this study provided evidence-based medical evidence support for the clinical application of dexmedetomidine.

## Data Availability Statement

The datasets presented in this study can be found in online repositories. The names of the repository/repositories and accession number(s) can be found in the article/supplementary material.

## Ethics Statement

The studies involving human participants were reviewed and approved by Hunan Provincial People's Hospital. The patients/participants provided their written informed consent to participate in this study.

## Author Contributions

YT is mainly responsible for the writing of the paper. JL and XH are mainly responsible for the design of the study and the inclusion of cases. HD and ST are mainly responsible for the evaluation of results and data statistics, and YZ is mainly responsible for the guidance of the whole research process. All authors contributed to the article and approved the submitted version.

## Funding

The current study was financially supported by following fund: Hunan Provincial Natural Science Foundation of China (Grant No. 2018JJ3296/Grant No. 2020JJ4404)/Fund of the Hunan Provincial Health Commission (Grant No. 20200758).

## Conflict of Interest

The authors declare that the research was conducted in the absence of any commercial or financial relationships that could be construed as a potential conflict of interest.

## Publisher's Note

All claims expressed in this article are solely those of the authors and do not necessarily represent those of their affiliated organizations, or those of the publisher, the editors and the reviewers. Any product that may be evaluated in this article, or claim that may be made by its manufacturer, is not guaranteed or endorsed by the publisher.
